# Aiding the resilience of cooperation through the use of network rankings

**DOI:** 10.1371/journal.pone.0313198

**Published:** 2024-11-26

**Authors:** Adam Lee Miles, Matteo Cavaliere, Guoli Yang

**Affiliations:** 1 Department of Computing & Mathematics, Manchester Metropolitan University, Manchester, Lancashire, United Kingdom; 2 Department of Physics, Informatics and Mathematics, University of Modena and Reggio Emilia, Modena, Italy; 3 Department of Big Data Intelligence, Advanced Institute of Big Data, Beijing, China; Teesside University, UNITED KINGDOM OF GREAT BRITAIN AND NORTHERN IRELAND

## Abstract

In many aspects of life on earth, individuals may engage in cooperation with others to contribute towards a goal they may share, which can also ensure self-preservation. In evolutionary game theory, the act of cooperation can be modelled as an altruistic act of an individual producing some form of benefit that can be utilised by others they are associated with at some personal cost. In various scenarios, individuals make use of information that they can perceive within a group to aid with their decision-making regarding who they should associate and cooperate with. However, cooperative individuals can be taken advantage of by opportunistic defectors, which can cause significant disruption to the population. We study a model where the decision to establish interactions with potential partners is based on the opportune integration of the individual’s private ability to perceive the intentions of others (private information) and the network position (ranking) of potential partners (public information). We find that there are rankings, such as degree and eigenvector, which can lead to a significant increase to the prosperity of the network, but this greatly increases the likelihood of a network succumbing to cheater invasion. Other rankings, such as betweeness, can instead lead to more stable resilient networks whilst also cultivating some degree of payoff. Our results highlight how commonly used network rankings can be utilised to aid with connection formation within networks and in turn can be utilised to improve the well-being of these networks, helping with stability and allowing for individuals to cultivate cooperation amongst each other. Private information should also continue to be considered when examining the dynamics of these networks as it appears to be a primary driver of encouraging individual agency.

## 1 Introduction

In various areas of life on earth, the act of cooperation between individual entities can be observed in many forms. The occurrences of cooperation can be observed within aspects of modern society, including within the global economy and in governments across the world [1–4]. The phenomena of cooperation is also found to occur in the natural world, including within animal social groups and cellular biology [5, 6]. When modelling these entities within the context of a network, it becomes possible to observe the finer dynamics of cooperation that may occur within these networks, such as how social capital, which could be considered as high connection count or providing access to other parts of the network, can have an effect on cooperative dynamics [7]. The processes of these networks can be expanded upon by considering how interactions between individuals can go on to affect their well-being and the decisions that they undertake, which are commonly considered within the field of evolutionary game theory [6].

Within the context of game theory, the act of cooperation is considered as an act where an individual chooses to incur some personal cost which will result in some form of benefit being produced for others who are associated with that individual [2]. For example, a worker bee in a colony may choose to put aside their own reproductive potential in order to aid with the protection of the colony. This act will indirectly raise the reproductive potential of other bees within the colony [6]. Ideally, other individuals in these kinds of scenarios will engage in like minded behaviour in order to contribute towards cooperative efforts within the group and improve general well-being. The costs incurred by individuals choosing to cooperate are ultimately outweighed by the benefits produced by others within the network [8]. The scale of these cooperative dynamics can range between interactions involving only two individuals to large interconnected networks of individuals where individual connection count can vary. This allows for representing different kinds of communities of varying scale and connectivity.

However, as has been observed in different games [6] and during historical events [9], the altruistic behaviour of these cooperative individuals can be taken advantage of by opportunistic defectors that may be present. In game theory, a defector or cheater is considered as an individual who chooses to engage in behaviour that potentially maximises their own payoff whilst ultimately being detrimental to individuals they are associated with, as they will not produce any benefits that others can take advantage of. These defectors will still take advantage of the benefits produced by other cooperative individuals, who are still incurring some cost. The game that is commonly used to illustrate this conflict is the prisoner’s dilemma [7]. Here, both players will have the option to either cooperate or to defect against their opponent. When examining the payoff matrix of this game, it becomes clear that defection is the more optimal strategy of the two available options, as it allows the player to potentially maximise their payoffs whilst also mitigating any losses. The conflict between cooperation and defection is commonly considered within evolutionary game theory [6], where the payoffs of these interactions can contribute to the fitness of individuals. Depending on how fitness is defined within a model, the fitness of individuals can gradually change over time [10], in response to changes in their local environment, can affect how they perform and can also impact the likelihood of reproducing.

Within these kinds of scenarios, individuals may utilise information that is available to them in order to aid with decision making [11]. In work carried out by Shirado et al. [12], the researchers conducted experiments where autonomous bots were utilised using various behaviours in order to aid with the rewiring of connections between individuals. Their findings showed that some bot strategies could be used to help steer individuals within the network towards cooperative acts. The assumption made within this paper is that a bot could concretely identify an individual as either a cooperator or a defector. In the real world outside the context of such a experimentation model, identifying the intentions of individuals could prove difficult and would likely require significant resources in order to aid with the classification of individuals, which could ultimately prove inaccurate over time as circumstances shift [13, 14].

One of the potential ways to address this is to consider existing methods of evaluating networks in order to aid with an individual’s decision-making when determining who they will associate with. In previous work carried out by Yang et al. [15], researchers explored how the presence of private and public information could influence the formation of connections as newcomers join a network of individuals. The connections that a newcomer may form during this process are limited by their choice of role-model, which is influenced by the fitness of individuals. The potential connections that a newcomer may form during this process is limited to the role-model and all individuals associated with the role-model. The choice of role-model will also influence the strategy that is adopted by the newcomer, which will determine how that newcomer will interact with its neighbours and, by extension, how that newcomer will contribute to the general well-being of the network. During decision-making, newcomers make use of two kinds of information, private and public. Private information is based on how well an individual can perceive if another individual is trustworthy. Newcomers within this model utilised the current degree average of the network to serve as some form of indication from what they can perceive from publicly observable information.

This paper expands upon this dynamic by allowing newcomers to utilise different kinds of network rankings to serve as public information in order to assist newcomers when determining who to associate with. The intuition here is that by allowing newcomers to use different methods of publicly perceiving the current state of the network, this will lead to generally different network structures. The effects of these changes to network structures can be analysed by examining the general well-being of networks as well as their resilience to the possibility of cheater invasion.

To evaluate the dynamics of these networks, we make use of an agent-based model which simulates multiple networks based on the currently defined configurations. During these simulations, newcomers will utilise different network rankings to provide indications regarding publicly observable information. We determine how utilising different network rankings can affect the resilience of these networks against cheater invasion and how some rankings are capable of kerbing the number of connections formed between individuals and therefore improving network stability against cheater invasion, a factor which ultimately has an effect on the short and long term fitness on individuals. These rankings are evaluated during several simulation sets where the priority of these indications are adjusted, affecting the likelihood of newcomers forming connections with potential neighbours. We find that there are specific rankings that are capable of resulting in networks where a degree of cooperation is cultivated amongst individuals whilst remaining resilient against cheater invasions.

## 2 Materials and methods

### 2.1 Computational model

The model utilised for this paper is based upon work previously carried out by Yang et al. [15] in order to explore how the use of different network rankings can potentially be used to increase network resilience against cheater invasions. In this previous work, the researchers consider networks where newcomers join at each step, where they will choose another individual to act as a role-model, which is a decision that is based upon the level of fitness of all individuals within the network. Following this, the newcomer will then adopt a strategy and then determine which other individuals they will be associated with.

Within this model, we utilise the prisoner’s dilemma in order to model interactions between individuals and to consider the conflict that can arise from cooperative scenarios [2]. The payoff matrix utilises parameter *b*, which determines the value of the benefits that are produced by individuals who choose to cooperate. Parameter *c* is used to determine the costs that are incurred when an individual chooses to engage in cooperation. Throughout all simulations, we assume that *b* = 9 and *c* = 8, which will adhere to the condition of *b* > *c* > 0. The payoff *P*_*i*_ of node *i* is determined by the sum payoff of an individuals interactions with all of its neighbours in the network. For example, assuming a cooperator has *K* cooperative neighbours and *J* defectors it is connected with, that individual will receive a payoff as described in Eq (1).

$$\begin{array}{c} P_{i}=K\left(b-c\right)+J\left(-c\right) \end{array}$$ (1)

For a cheater, assuming a cooperator has *K* cooperative neighbours and *J* defectors, that individual will instead receive a payoff as described in Eq (2).

$$\begin{array}{c} P_{i}=K\left(b\right)+J\left(0\right) \end{array}$$ (2)

Parameter *δ* is used to determine the level of selection strength, which increases the influence that an individuals payoff will have when a newcomer is selecting a role-model. In conjunction with *P*_*i*_, this parameter is used to calculate the fitness (*f*_*i*_) of individuals on the network, which is described in Eq (3). During simulations, we assume *δ* = 0.01 in order to establish medium selection strength when calculating this value for individuals.

$$\begin{array}{c} f_{i}={\left(1+\delta \right)}^{Pi} \end{array}$$ (3)

The model utilised in this paper is adapted from previous works [15], where networks are simulated over 10^8^ steps to determine how particular network configurations would affect network dynamics. Where this model differs here we simulate multiple networks using the same configurations and then observe how the network performs when subjected to a forced cheater invasion. By running simulations where a large series of networks are observed during cheater invasion, this will allow for a comprehensive examination of how networks will generally perform under certain conditions. For all configurations that are considered during simulations, 1000 networks will be ran for each set. At each step, newcomers join a network of fixed size, and will choose a role-model based on individual level of fitness, similar to previous works [15].

As the model is simulated, each network will first be initialised using Erdős-Réyni [7, 16] in order to randomly generate the starting structure, with 30% of all possible edges being created between *N* = 100 nodes.

Following this choice, a newcomer will then utilise private and public information in order to evaluate potential neighbours, which includes the role-model and any individuals who share a connection with the role-model, and then the newcomer will determine which connections it will create as it joins the network.

**Fig 1:**
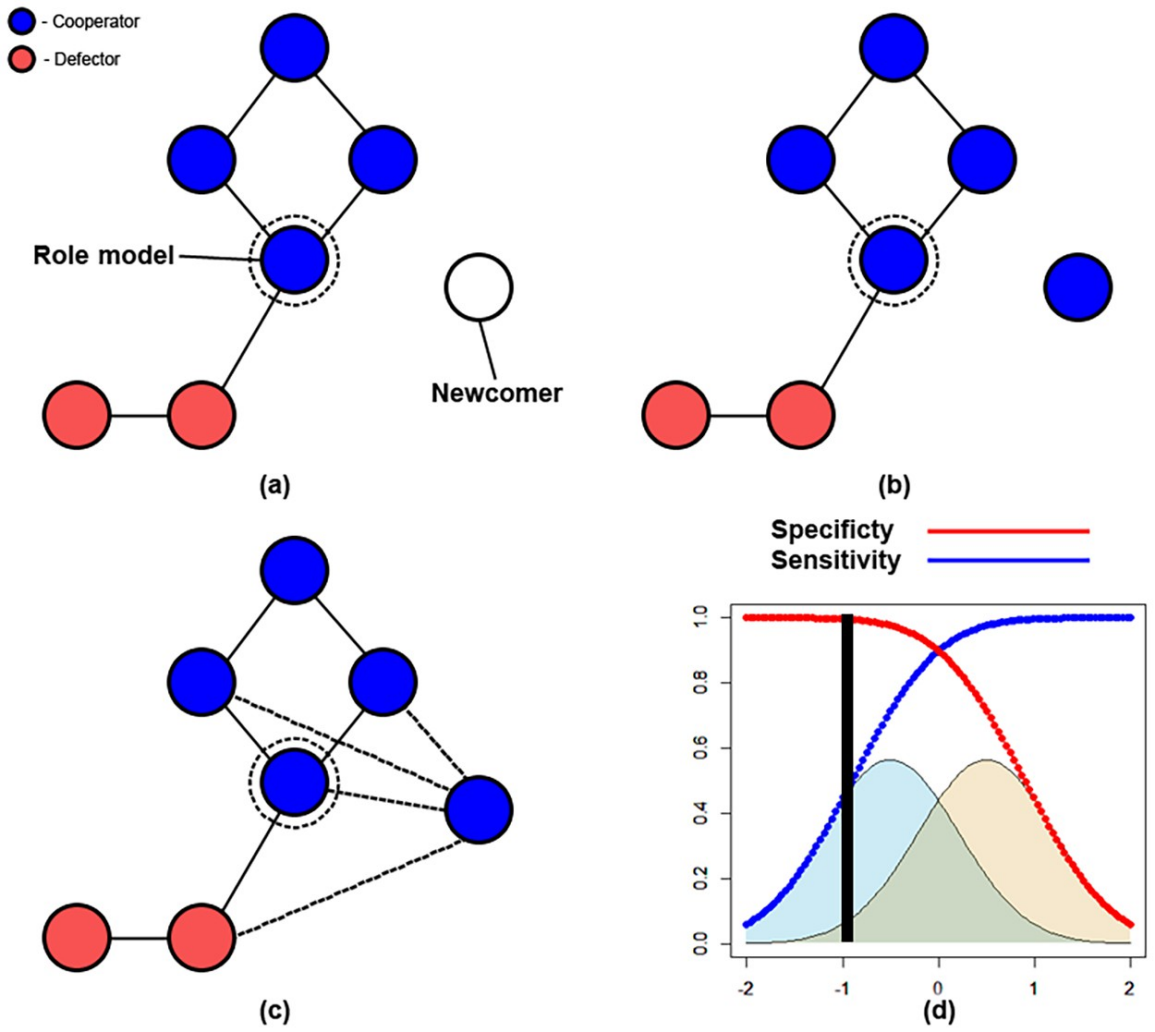
An illustration of the process that is undertaken as a newcomer joins the network at each step during the simulation. Firstly, a newcomer will select an existing node to act as its role-model, which is influenced by node fitness and *δ* = 0.001 (a). Following this, the newcomer will adopt a strategy by copying the role-model (b). Lastly, the newcomer will then proceed to choose which connections to form between the role-model and the role-models neighbours, which is done by making use of available public and private information (c) [15]. To model private information, two Gaussian distributions are used to represent an individuals ability to identify either cooperators or defectors respectively (d).

At each step during a network simulation, a newcomer will be integrated into the network (see Fig 1). First, a random individual is selected to act as the newcomer’s role-model (see Fig 1a). The selection is influenced by individual level of fitness, as described in Eq (3). The higher this value, the more likely an individual will be selected to act as a newcomer’s role-model. Following this, the newcomer will then adopt the strategy of its chosen role-model (see Fig 1b). Following this, private and public information are then utilised by the newcomer in order to determine which connections it will form between the role-model and any individual the role-model is currently connected with (see Fig 1c). After a newcomer’s connections have been established, the newcomer will then engage in a round of the prisoner’s dilemma with all individuals it has chosen to form a connection with, which will determine its total payoff as described in Eqs (1) or (2) based on its selected strategy.

### 2.2 Private information

As in previous works [15, 17], two Gaussian distributions (see Fig 1d) are utilised to model a newcomers ability to determine the intentions of a potential neighbour as private information. Both distributions are created with a variance of *σ*^2^ = 0.5 and both peaks are set 1 apart from each other, with a *μ* of -0.5 for *ϕ*_*c*_ and a *μ* of 0.5 for *ϕ*_*d*_. The use of these two overlapping distributions *ϕ*_*c*_ and *ϕ*_*d*_ are used in order to model an individual’s imperfect ability to differentiate between potential cooperators and defectors respectively. For each node *x* (which can be either the role-model or any other nodes connected with the role-model) where a connection can be formed, a value is either sampled from *ϕ*_*c*_ if node *x* is a cooperator or *ϕ*_*d*_ if node *x* is a defector. Should the *decision* *threshold* *τ* exceed the sampled value, then the private information will indicate that a connection should be made with node *x*. Otherwise, private information will instead indicate that a connection should not be created with node *x*. Increasing the value of *τ* across simulations will increase the likelihood that private information will indicate that a connection should be made. Whilst this will mean that newcomers will be more likely to form more connections with other nodes, this will also mean that the likelihood of connecting with a defector will also increase (see Fig 1d).

### 2.3 Individual network rankings as public information

In previous works [15, 17], public information is modelled as a newcomer evaluating the number of connections a potential neighbour possesses against the current network average. If the number of connections exceeded the network average, then public information would indicate that a connection should be formed with that node. Here, we expand upon this by considering additional methods of public information by evaluating a potential neighbour based on information regarding network topology. Here, we utilise the *degree* *centrality* method [18], as described below, in order to understand how utilising additional methods of network evaluation can lead to changes in the dynamics of these cooperative networks. In addition to connection average, we also consider here two other common rankings used to analyse networks in order for newcomers to evaluate potential neighbours. Firstly, *betweeness* *centrality* [19] which considers the traversability that a node offers within a network and *eigenvector* *centrality* [20] which considers the social capital of not just a particular node but also of its local neighbours [7]. In addition to these proposed rankings, we also consider how the network average of these values are utilised when evaluating individuals. To further consider the use of additional methods as part of node decision-making, we also consider the possibility of if the signal indicated from public information is randomly determined rather than being based on a defined method. In conjunction with the random method, parameter *rc* is used to determine the probability that public information will indicate positively when newcomers are utilising this method of node evaluation. With these implemented into the model, the following rankings are then considered:

- Degree High (Deg. Hi.)—evaluates if an individuals degree centrality [18] exceeds the network average, if so, public information will indicate a connection should be formed.
- Degree Low (Deg. Lo.)—evaluates if an individuals degree centrality [18] falls below the network average, if so, public information will indicate a connection should be formed.
- Betweeness High (Bet. Hi.)—evaluates if an individuals betweeness centrality [19] exceeds the network average, if so, public information will indicate a connection should be formed.
- Betweeness Low (Bet. Lo.)—evaluates if an individuals betweeness centrality [19] falls below the network average, if so, public information will indicate a connection should be formed.
- Eigenvector High (Eig. Hi.)—evaluates if an individuals Eigenvector centrality [20] exceeds the network average, if so, public information will indicate a connection should be formed.
- Eigenvector Low (Eig. Lo.)—evaluates if an individuals Eigenvector centrality [20] falls below the network average, if so, public information will indicate a connection should be formed.
- Random, *rc* = 0.8 (Rand.8.)—public information indicates that a connection should be formed with a probability of *rc* = 0.8.

### 2.4 Information based decision-making

The indications that are provided from private and public information (see Sections 2.2 and 2.3) are then utilised by newcomers to determine if they will form a connection with a node *x*, which will either be the role-model or any of the nodes that are currently connected to the role-model (see Fig 1c). The indications for whether or not a connection should be formed are described in Sections 2.2 and 2.3. Both of these indications provided by private and public information are weighted in order to consider the likelihood of a connection being formed with node *x* should only one of these indicate positively. Parameter *p* will determine the likelihood that a connection will be formed with node *x* when only public information indicates a connection should be made. Similarly, parameter *q* will determine the likelihood that a connection will be formed with node *x* when only private information indicates positively. Throughout simulations, *p* and *q* are adjusted in order to assign varying weight to each type of information.

Given the node *x*, the chosen role-model or a neighbour of the chosen role-model and the indications from public and private information, the newcomer decides whether to connect with a node *x* in the following way:

- If both public and private information indicate a connection should be made, form connection with node *x*
- If both public and private information indicate a connection should not be made, reject connection with node *x*
- If public information indicates to make a connection but private information does not, a connection with node *x* will be created with probability *p*
- If private information indicates to make a connection but public information does not, a connection with node *x* will be created with probability *q*

Following a newcomer deciding which connections it will form within the role-model’s local neighbourhood (Fig 1c), the network is then updated. Following this, an individual is randomly selected to be replaced by the newcomer, which keeps the population size fixed as in the Moran process [6]. Following this, the currently selected network ranking is recalculated for all individuals and for the network average, which will be utilised by the next newcomer that joins the network.

### 2.5 Perturbation & network evaluation

**Fig 2:**
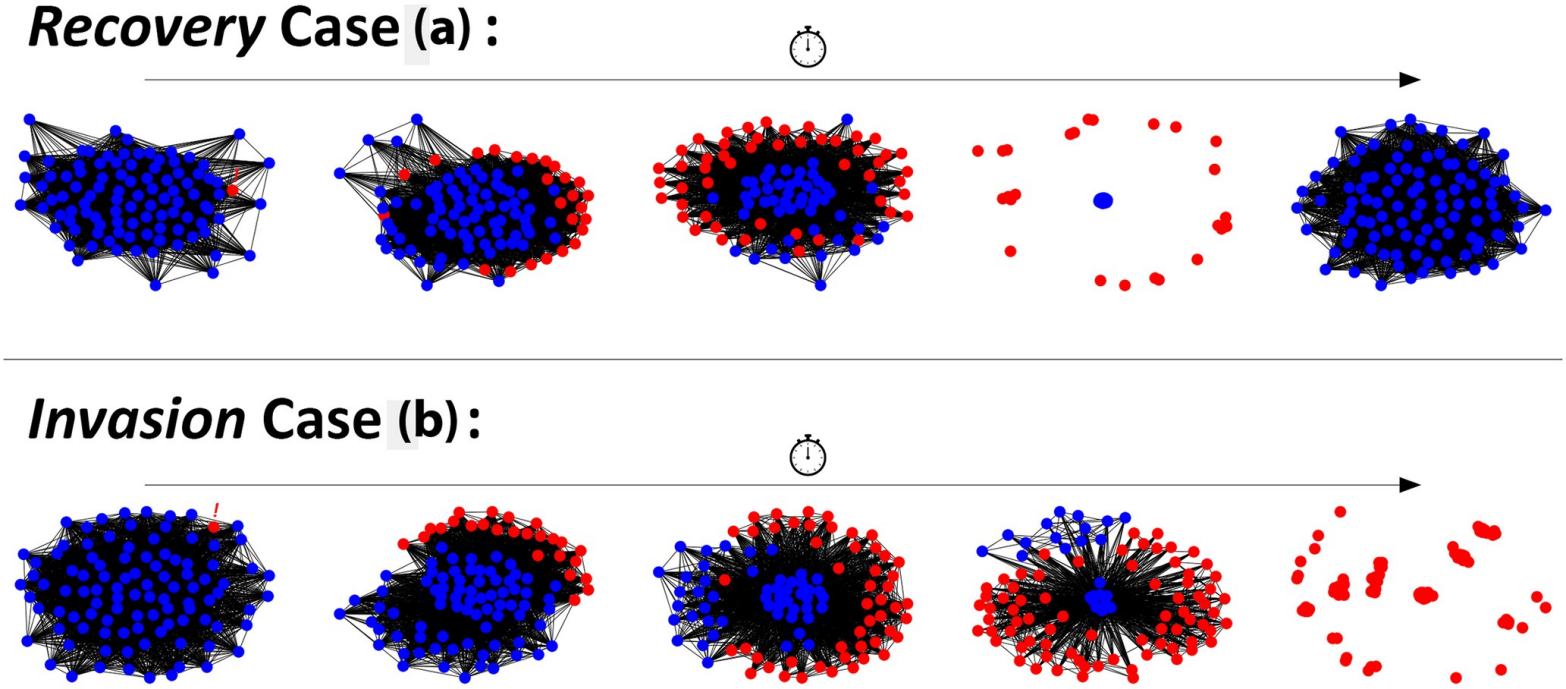
After 10,000 steps, an individual is randomly selected to change its strategy to cheating, a methodology adapted from previous works [15]. The network will continue to be simulated whilst both strategies are present. Should the network progress to a state where only cooperators are present, this will be considered as a *recovery* (a). Otherwise, if a network progresses to the point where only cheaters remain, this will considered as a successful *invasion* (b). Snapshots produced via NetworkX for Python.

As described in Section 2.1, newcomers that join the network will adopt the strategy of its chosen role-model. Following the first 10,000 rounds of a network (see Fig 2a), an individual is randomly selected to mutate, changing its strategy to defection. We allow a network to progress for 10,000 rounds in order to allow for a sufficient amount of time for a network to settle into its general state based upon the current parameters that have been set for the simulation. Controlling the step of mutation allows for controlled observations of multiple cheater invasions, which should provide some insight into how the use of certain network rankings can aid with network resilience to cheater invasion. Within the model, we consider an invasion as resolved should the network progress to a state where only one strategy remains following the start of an invasion.

To evaluate the use of certain rankings for public information, we observe and log the outcomes of these cheater invasions. Following the start of an invasion, should the network return to a state where only cooperative individuals remain, we consider this as an instance of a *recovery* (see Fig 2a). Should a network instead progress to a state where only defectors remain, we consider this as an instance of an *invasion* (see Fig 2b). The number of these occurrences are logged for each simulation set will be considered when evaluating the general well-being of these networks. A high number of recoveries will suggest that a network is resilient to potential cheater invasion whereas a low number of recoveries will suggest that the conditions of a network make it susceptible to the propagation of defection across the network. In addition to these, we also consider the average number of steps that occurred for these invasions to resolve and are divided into their respective outcomes. For recoveries, the average number of steps to reach this point will be considered as *recovery* *time*. For successful invasions, the average number of steps to reach this point will be considered as *invasion* *time*. These additional metrics will also allow for determining if particular network configurations are capable of speeding up the average rate of recovery in networks or if they are capable of slowing down the rate of cheater invasions.

Once a network invasion has resolved, the outcome of the current network is logged, and simulation data is updated as required. The model will then initialise and simulate a new network based on the current configurations. This will continue until all 1000 networks for a set of parameters have been simulated. During the simulation of these networks and in addition to the previously described metrics for evaluating the outcomes of invasions, several additional metrics are also utilised. Pre-invasion payoff is used to determine the average level of payoffs of individuals across networks at the step just before an existing individual is chosen to mutate into a cheater. Pre-invasion connections is used to determine the average number of connections that are present within networks at the same point as the pre-invasion payoff. We also consider the payoff ratio of individual *i* at the step just before a cheater invasion begins, which is calculated as a normalised value based upon the maximum potential payoff that could be obtained by a cooperative individual.

## 3 Results

To evaluate how the usage of different network rankings can affect the emergence of cooperation and the resilience of networks against cheater invasion, several series of simulations are carried out. As previously described, each set of simulations carried out will simulate 1000 random networks that each utilise the same parameters in order to obtain a concise idea of how particular configurations can affect networks. These simulations are separated into four groups, based on the balance of information prioritisation, which is adjusted via the *p* and *q* parameters within the model (see Section 2.4). For each set, parameter *τ* is also adjusted to determine the decision threshold of newcomers when evaluating potential neighbours as part of private information, which is adjusted in increments of 0.2 with a minimum of −2 and a maximum of 2.

### 3.1 Networks favouring less connected individuals leads to greater stability

For the first series of simulations, networks were observed when prioritisation for both types of information are low (*p* = 0.25, *q* = 0.25), meaning newcomers are less likely to form new connections when at least one information type does not indicate a connection should be formed.

**Fig 3:**
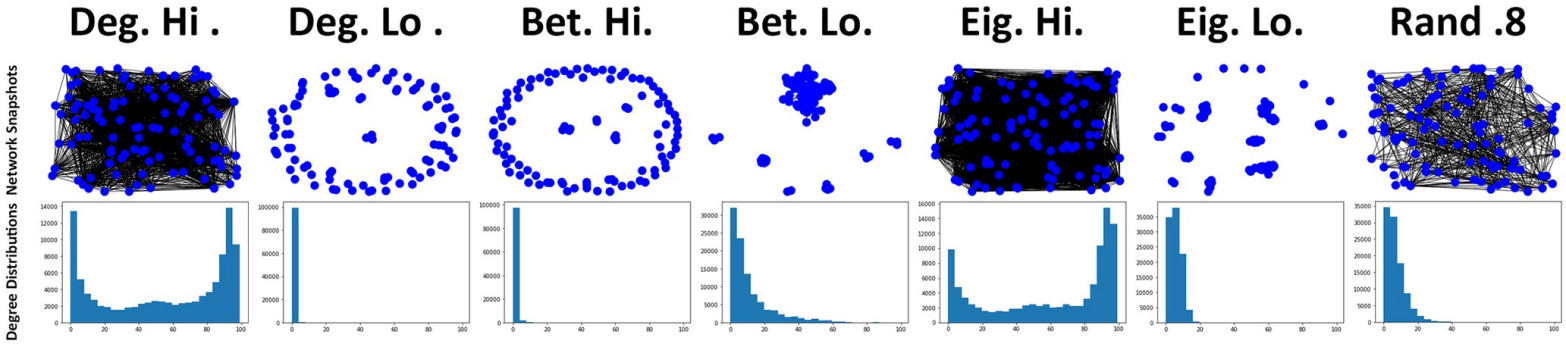
Networks rankings favouring more connected individuals become more densely interconnected even when signals are low, reflecting previous works [21]. Top row consists of visual snapshots of networks at the step and bottom row consists of degree distribution plots visualising the number of connection counts of individuals, both at the step just before an existing individual is selected to mutate into a cheater where *τ* = 1.6 where prioritisation of both information types are low (*p* = 0.25, *q* = 0.25). Snapshots produced via NetworkX libraries for Python and distribution plots produced via Jupyter notebook.

**Fig 4:**
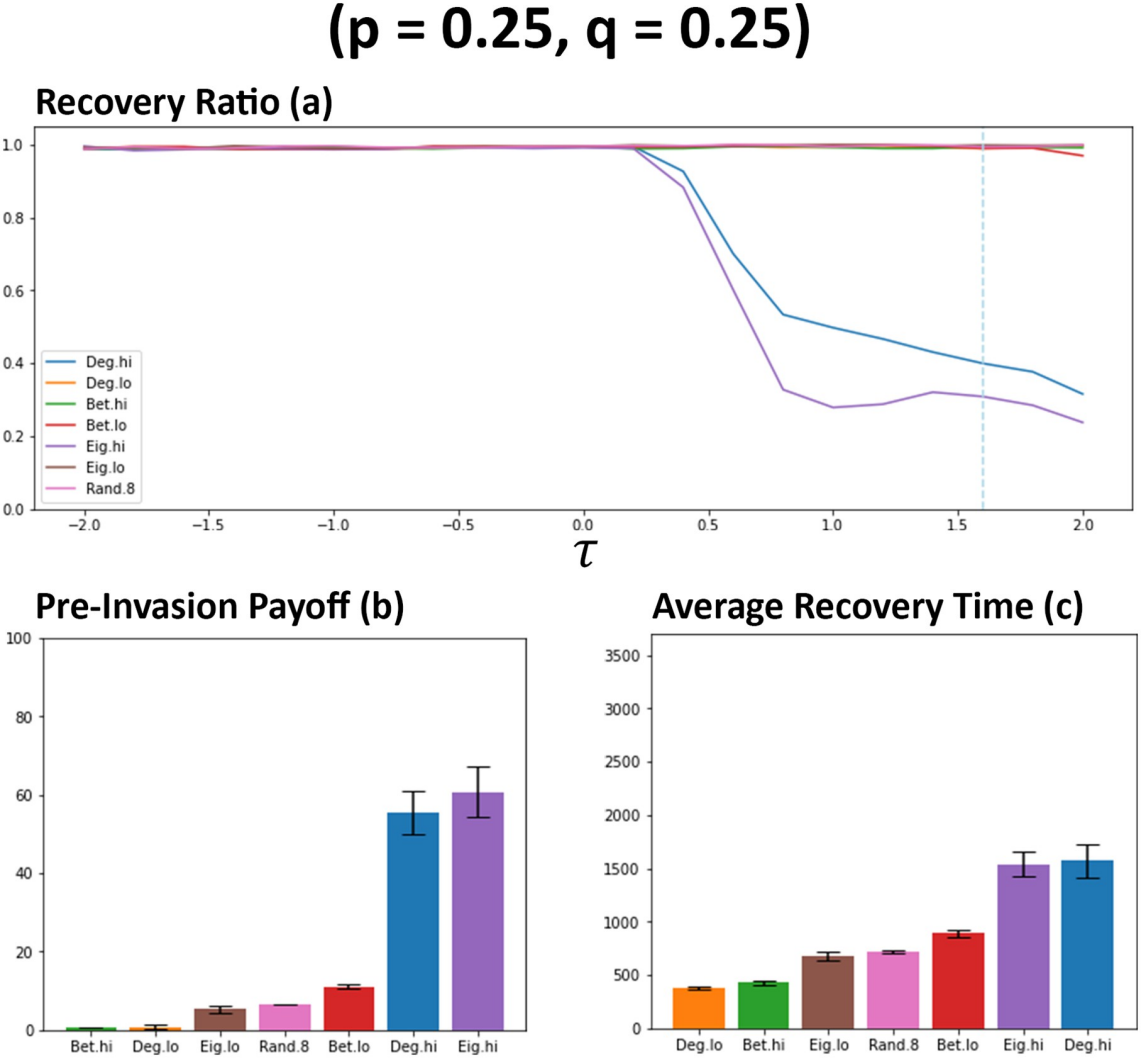
Networks favouring more connected individuals results in a significant decrease to the likelihood of network recovery. Plot (a) visualises the metrics (see Section 2.5) of ratio of recoveries that occurred for each network rankings utilised by newcomers. Plots (b) and(c) visualises the average payoffs of individuals before an induced invasion and the average number of rounds that occurred when recoveries were successful respectively. Both (b) and (c) are sampled from networks where *τ* = 1.6, as indicated by the blue dashed line on plot a. Data obtained by running the model with 1000 networks simulated per simulation set where prioritisation of both information types are low (*p* = 0.25, *q* = 0.25). *τ* represents the decision threshold. Plots produced via Jupyter Notebook.

As can be observed in Fig 3, the choice of network ranking utilised by individuals has an impact on the number of connections that are formed between individuals. This becomes most apparent when *τ* > 0 and generally remains the case for other observed rankings in all simulations that were carried out. Newcomers that utilise degree (Deg. hi) and eigenvector (Eig. hi) rankings that favoured more interconnected individuals results in a significant increase in average connectivity and the occurrences of bimodal distributions when observing connectivity at the point of invasion (see Fig 3). All other rankings utilise, of which most favour less connected individuals, result in significantly less connections between individuals, with betweeness low (Bet.lo) emerging as the better of these methods with moderate gains in connectivity. The observations regarding connectivity are also tightly aligned with that observed for the average payoffs (see Fig 4b). As connectivity increases, so too does the average payoff that newcomers generally receive amongst their cooperative neighbours. However, this significant increase to payoff for some rankings does come with a heavy price of significantly less recoveries occurring (see Fig 4a). It would appear that with the significant increase of connectivity and payoff comes a significant decrease to the likelihood that a network will recover from a cheater invasion, which reflects what has been observed in previous works [21]. In the circumstance where defectors successfully invade a network, resulting in no remaining cooperators, this essentially renders any gains in payoff before an invasion moot. Of the other methods, betweeness low (Bet. lo) performs the best with some increases to payoff (see Fig 4b) at a minor cost to recovery ratio (see Fig 4a). The choice of rankings also has a noticeable impact on the average recovery time of networks. Aligning with observations regarding connectivity and payoff (see Figs 3 and 4b), average recovery time increases when the utilised network ranking favours more interconnected individuals (see Fig 4a). Other utilised rankings appear to only result in minor increases when *τ* > 0. These suggest that not only are these networks are more resilient to cheater invasion, but can also resolve the invasion quicker (see Fig 3).

### 3.2 Betweeness offers strong resilience to invasion as private information is prioritised

For the next series of simulations, networks are adjusted so that private information is prioritised over public information (*p* = 0.25, *q* = 0.75), meaning that newcomers are more likely to form connections when only private information indicates positively.

**Fig 5:**
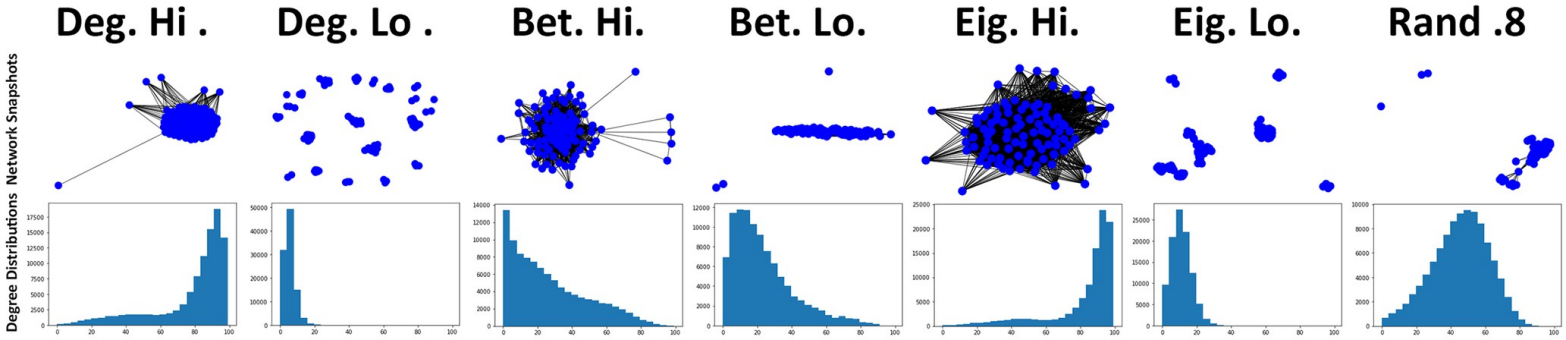
Connectivity of networks increases as private information is prioritised, although some methods, such as betweeness, appear to be capable of kerbing the number of connections formed, which contributes towards network stability. Top row consists of visual snapshots of networks at the step and bottom row consists of degree distribution plots visualising the number of connection counts of individuals, both at the step just before an existing individual is selected to mutate into a cheater where *τ* = 1.6 where private information is prioritised (*p* = 0.25, *q* = 0.75). Snapshots produced via NetworkX libraries for Python and distribution plots produced via Jupyter notebook.

**Fig 6:**
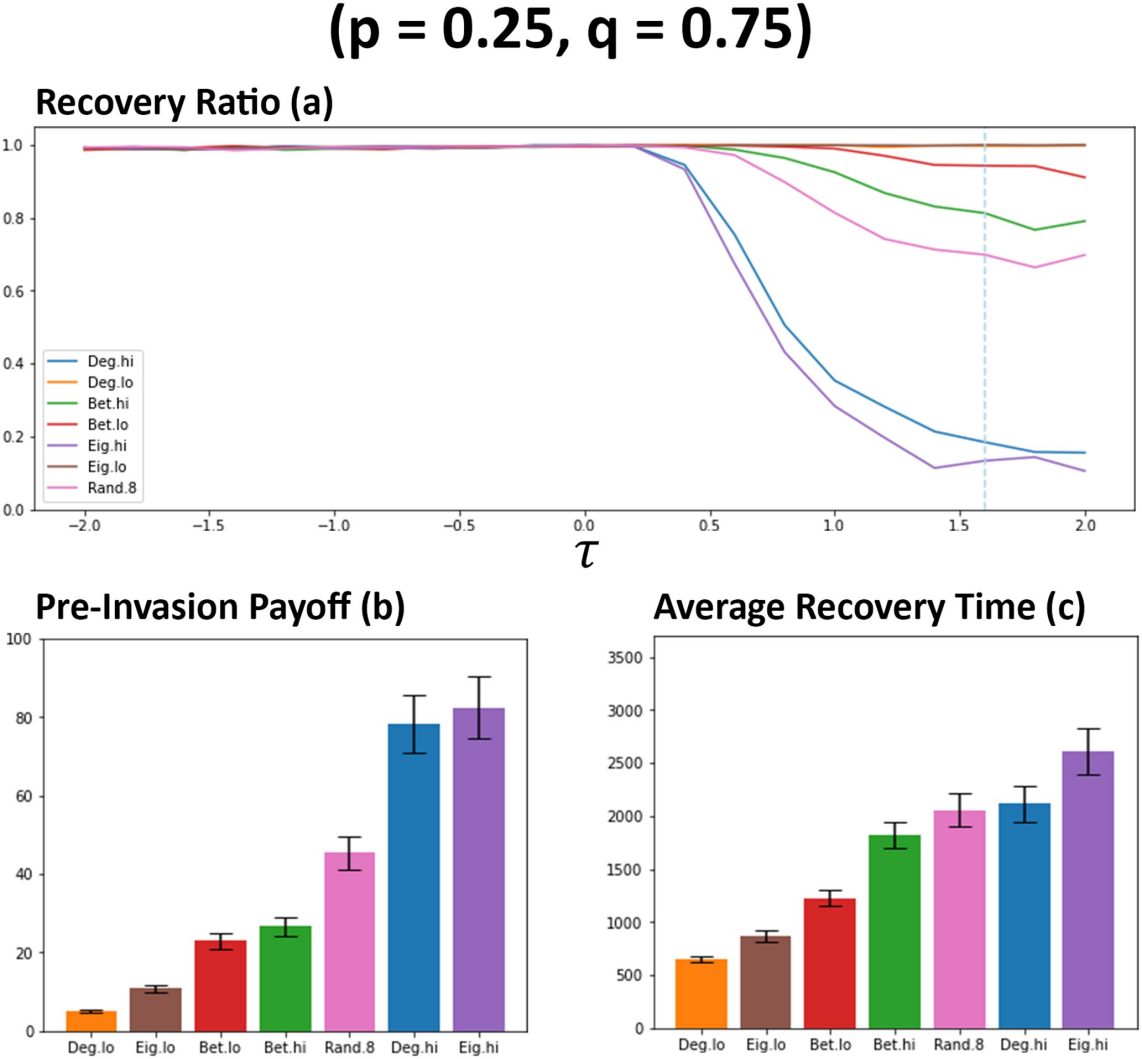
General increases to network connectivity and payoff observed as prioritisation for private information is increased in networks, along with potential decreases to recovery ratios. Plot (a) visualises the metrics (see Section 2.5) of ratio of recoveries that occurred for each network ranking utilised by newcomers. Plots (b) and (c) visualises the average payoffs of individuals before an induced invasion and the average number of rounds that occurred when recoveries were successful respectively. Both (b) and (c) are from networks where *τ* = 1.6, as indicated by the blue dashed line on plot a. Data obtained by running the model with 1000 networks simulated per simulation set where prioritisation of both information types are low (*p* = 0.25, *q* = 0.75). *τ* represents the decision threshold. Plots produced via Jupyter Notebook.

When the signal of *q* is increased, the general level of network connectivity also increases when compared against previous observations (see Fig 5). Similar to previous observations (see Fig 3), networks favouring more connected individuals (Deg. Hi & Eig. Hi) continue to garner the highest level of connectivity within networks Fig 5. When *q* is increased, the number of connections formed when there is a random chance (*rc* = 0.8) of public information indicating a connection should be formed significantly increases, overtaking other methods it was previously similar to (see Fig 5). Betweeness high (Bet. Hi) also results in an increase to connectivity under these circumstances, overtaking betweeness low (Bet. lo). As with previous observations, these changes to connectivity are also tightly reflected in the average payoffs of individuals within these networks (see Fig 6b), with individuals generally cultivating a greater payoff with more connections being present within networks. With this greater level of payoff comes the greater impact on the general recovery ratios of these networks (see Fig 6a), with degree high (Deg. hi) and eigenvector high (Eig. hi) methods being more affected than previous observations (see Fig 4a). Although random (*rc* = 0.8) performs better here, this also comes with a greater risk of succumbing to cheater invasions than other methods it was previously aligned with (see Fig 4). When private information has a stronger signal via *q*, there is a noticeable shift in the general level of connectivity of networks utilising betweeness high (see Fig 5) which by extension leads to some increases in payoff at a cost to the recovery ratio (see Fig 6a and 6b). Betweeness low is able to maintain a similar level of payoff whilst also being able to avoid a large decrease to recoveries. When private information is prioritised, a hierarchy emerges in terms of average recovery time (see Fig 6c). Degree and Eigenvector methods continue to result in generally longer recovery times when favouring more connected individuals. Interestingly, at points during observations, the random method (*rc* = 0.8) can actually result in longer recovery times than networks where there were significantly less recoveries, which may further suggest randomly selecting individuals can also potentially compromise the well-being of these networks [22]. Betweeness low (Bet. lo) attains a quicker recovery rate than betweeness high (Bet. hi), which could be explained by the differences in connectivity (see Fig 5). Methods favouring less connected individuals appear to result in fairly quick resolutions to cheater invasions (see Fig 6c), although this hints at low levels of connectivity in these networks.

### 3.3 Prioritising public information extends the regime of cooperation collapse

Following on from previous simulations, networks are then adjusted so that public information is now prioritised over private information (*p* = 0.75, *q* = 0.25), meaning that newcomers will be more likely to form connections when only public information indicates a connection should be formed with a potential neighbour.

**Fig 7:**
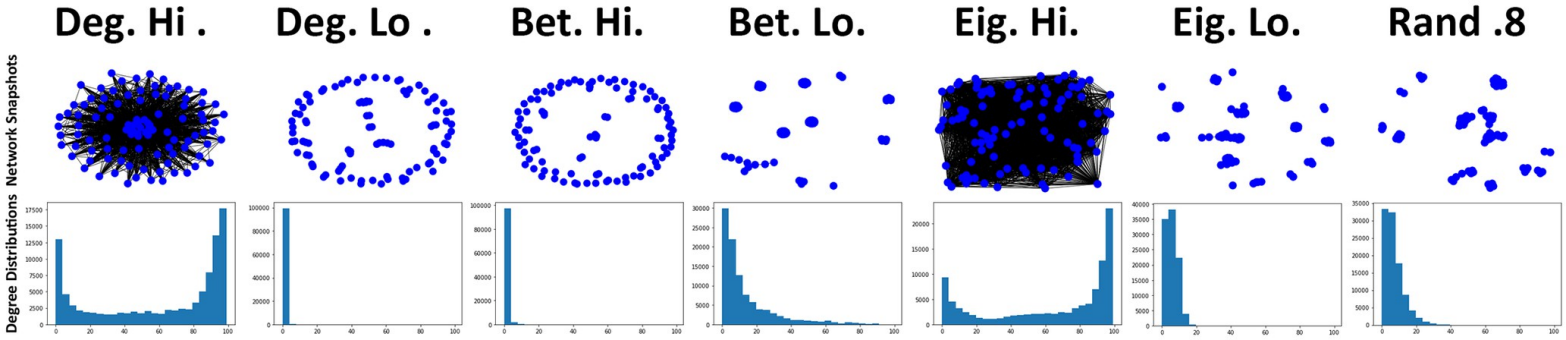
Prioritising public information leads to increases in network connectivity, but is generally lower than when private information is prioritised. Top row consists of visual snapshots of networks at the step and bottom row consists of degree distribution plots visualising the number of connection counts of individuals, both at the step just before an existing individual is selected to mutate into a cheater where *τ* = 1.6 where public information is prioritised (*p* = 0.75, *q* = 0.25). Snapshots produced via NetworkX libraries for Python and distribution plots produced via Jupyter notebook.

**Fig 8:**
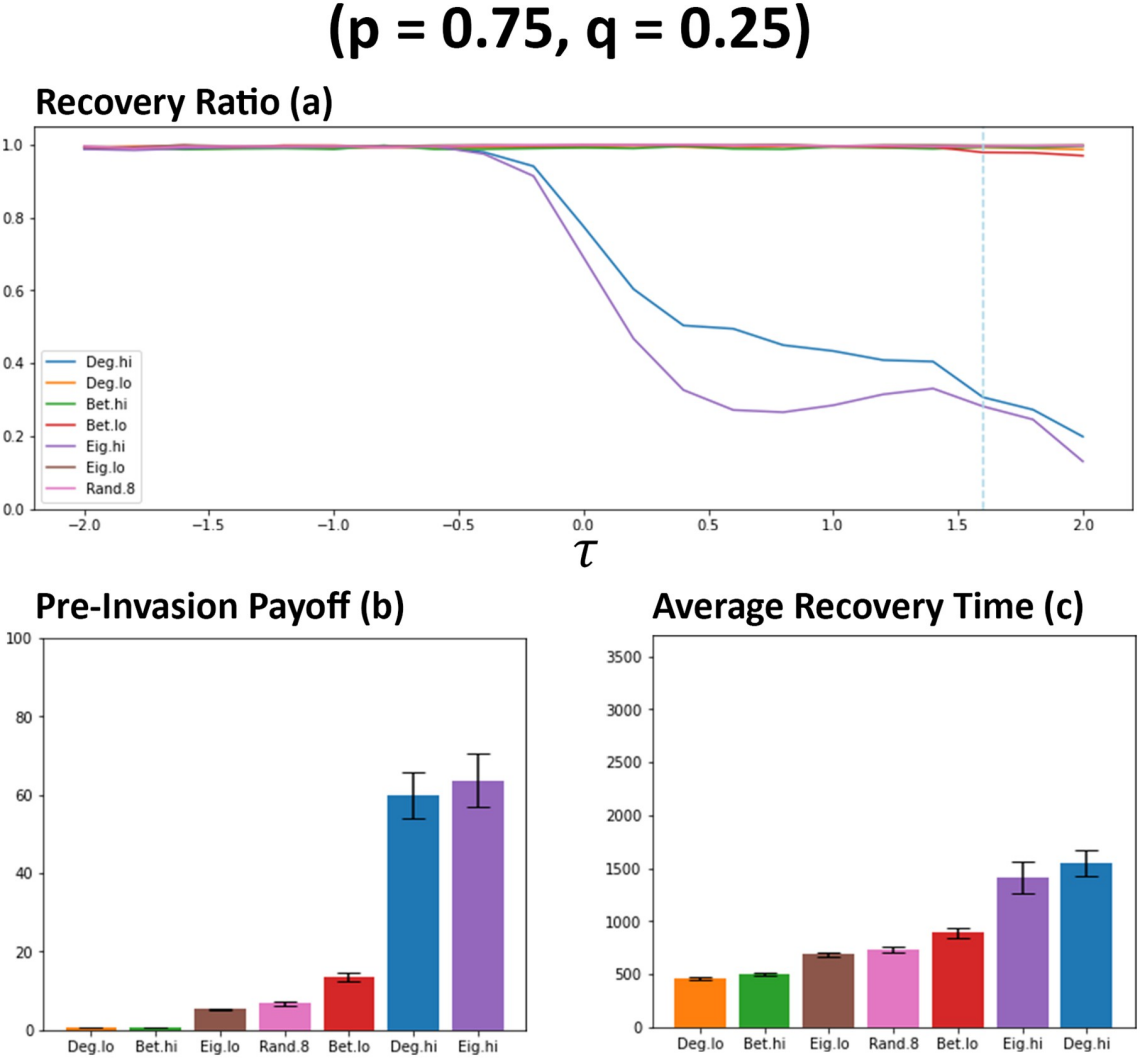
Prioritising public information results in less recoveries occurring at lower values of *τ* when compared against previous networks (see Fig 6). Plot (a) visualises the metric (see Section 2.5) of ratio of recoveries that occurred for each network ranking utilised by newcomers. Plots (b) and (c) visualises the average payoffs of individuals before an induced invasion and the average number of rounds that occurred when recoveries were successful respectively. Both (b) and (c) are from networks where *τ* = 1.6, as indicated by the blue dashed line on plot a. Data obtained by running the model with 1000 networks simulated per simulation set where prioritisation of both information types are low (*p* = 0.75, *q* = 0.25). *τ* represents the decision threshold. Plots produced via Jupyter Notebook.

When only *p* is increased, the number of connections formed between individuals decreases when compared against simulations where *q* was increased (see Fig 7). This, by extension, also leads to a noticeable decrease in the payoffs cultivated between individuals (see Fig 8b). This initial observation suggests that individuals are less likely to form connections when prioritising available public information rather than when they prioritise their individual ability to differentiate between individuals, something which has also been observed in previous works [11]. The point where recovery ratio begins to suffer occurs at lower values of *τ* (see Fig 8a) when compared against previous observations (see Fig 6a). The point where this initial decrease begins to occur aligns with the point where the initial increases in payoffs are observed. Degree high (Deg. hi) and eigenvector high (Eig. hi) continue to struggle with network resilience beyond *τ* > 0, although the number of recoveries for these networks is somewhat higher than previous observations where private information is prioritised (see Fig 6a). The impact on network recovery where other methods are utilised is minimal, the results suggest this may be related to the low connection count. Average recovery time (see Fig 8c) is comparably overall lower than previous simulations (see Fig 6c), with methods favouring less connected individuals typically resulting in a fairly quick turn-around to resolve a cheater invasion.

### 3.4 Rankings kerb network connectivity as strong signals are present

For the last series of simulations, networks are readjusted so that both types of information are prioritised (*p* = 0.75, *q* = 0.75), meaning that newcomers are highly likely to form new connections with potential neighbours when at least one information type indicates a new connection should be made.

**Fig 9:**
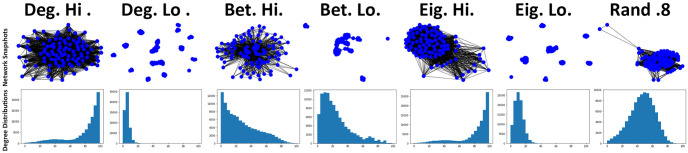
Strong signals from both public and private information results in greatest levels of connectivity, with some methods effective at reining this in. Top row consists of visual snapshots of networks at the step and bottom row consists of degree distribution plots visualising the number of connection counts of individuals, both at the step just before an existing individual is selected to mutate into a cheater where *τ* = 1.6 where prioritisation of both information types are high (*p* = 0.75, *q* = 0.75). Snapshots produced via NetworkX libraries for Python and distribution plots produced via Jupyter notebook.

**Fig 10:**
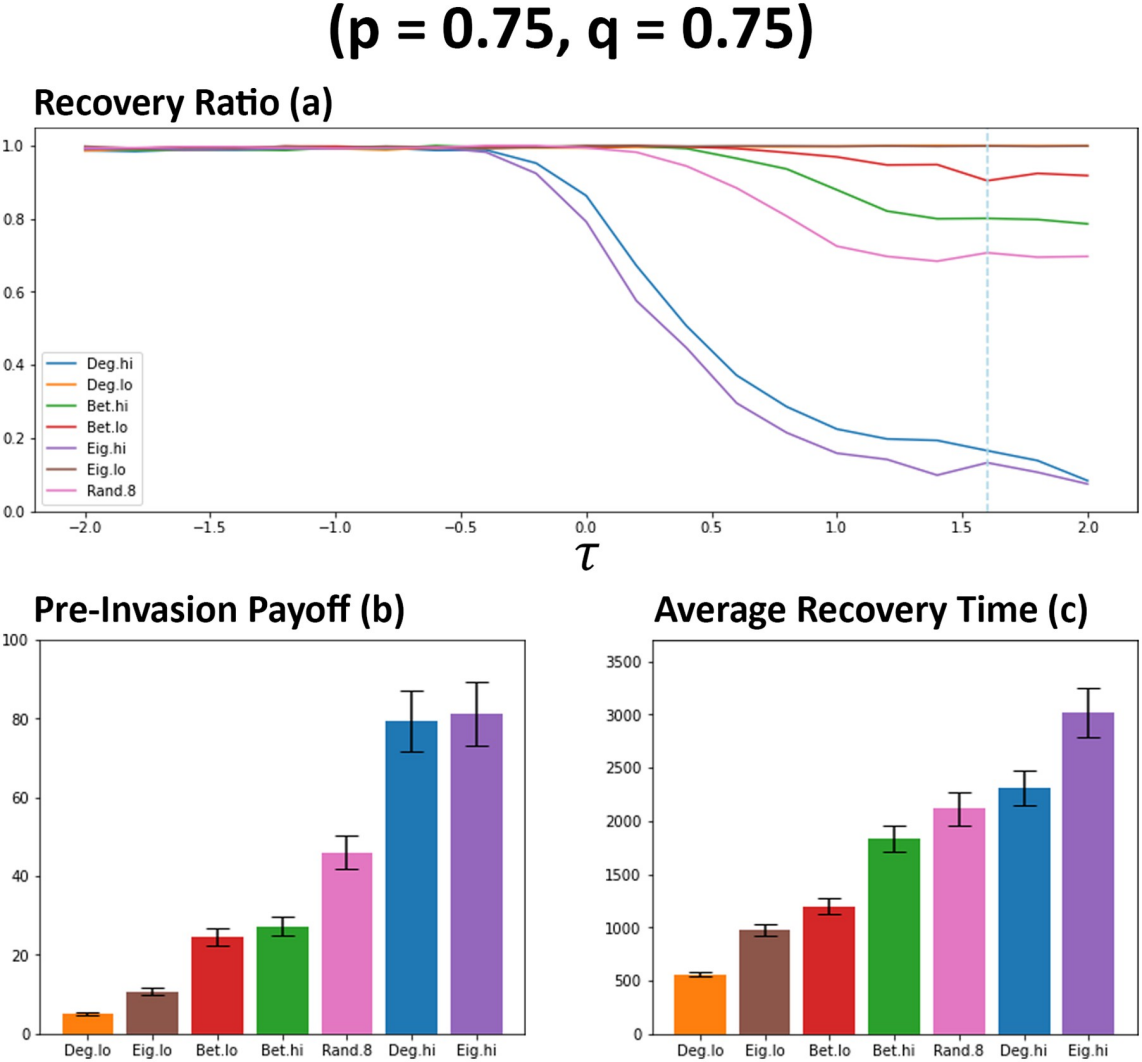
Strong information signals leads to the greatest impact to recovery rates (a), although some methods can mitigate the effects of this. Plot a visualises the metric (see Section 2.5) of ratio of recoveries that occurred for each network ranking utilised by newcomers. Plots (b) and (c) visualises the average payoffs of individuals before an induced invasion and the average number of rounds that occurred when recoveries were successful respectively. Both (b) and (c) are from networks where *τ* = 1.6, as indicated by the blue dashed line on plot a. Data obtained by running the model with 1000 networks simulated per simulation set where prioritisation of both information types are high (*p* = 0.75, *q* = 0.75). *τ* represents the decision threshold. Plots produced via Jupyter Notebook.

When both *p* and *q* are increased, this results in the highest level of connectivity observed within these simulated networks (see Fig 9), which by extension, also results in a significant increase to the average payoffs of individuals (see Fig 10b). As with previous simulations where only *q* = 0.75, the random method (Rand .8) results in significantly more connections under these circumstances. This, along with other changes here, illustrates the impact of private information as connectivity greatly increases when the signal from *q* is high, even when there is a high chance of public information indicating a connection should be formed. The initial increases in connectivity also appear to occur for lower values of *τ* as in previous simulations, which suggests that this may be caused by public information playing a stronger role in decision-making. As previously observed (see Fig 6a), this increase to connectivity and payoffs does come at a cost of network well-being, with the lowest recovery ratios observed here out of all simulation sets (see Fig 10a). Betweeness low (Bet. lo) results in somewhat more recoveries than betweeness high (Bet. hi) (see Fig 10a) for a minor impact to cultivated payoffs (see Fig 10b). Random networks (Rand .8) are able to attain a greater degree of payoff (see Fig 10b), but this does carry a greater risk of network collapse (see Fig 10a). Networks where more connected individuals are favoured result in a significantly low number of recoveries for the highest values of *τ*. Recovery times also appear impacted by these high signals, with the longest potential average recovery times observed here (see Fig 10c). Eigenvector high (Eig. hi) results in the longest recovery times, with degree high (Deg. hi) and random (Rand .8) resulting in similar high recovery times.

**Fig 11:**
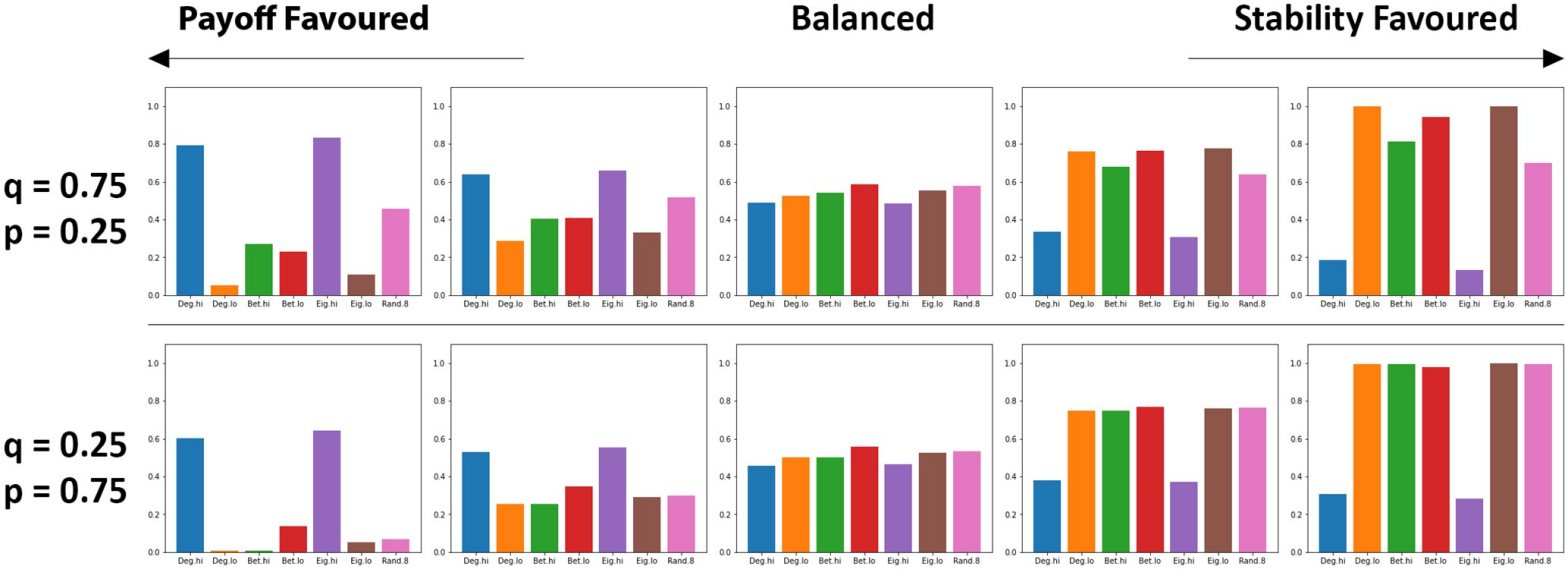
The trade-off between stability and prosperity can be controlled by individual rankings. The use of network rankings as public information can result in significant gains in payoff (here calculated as the normalised average payoffs of individuals) whilst other methods are significantly more robust in terms of stability (represented here using recovery ratio). When considering a balance of these factors, betweeness low emerges as a method offering moderate gains in payoffs and robust resistance against potential cheater invasions. Each plot value calculated here considers the level of prosperity against recovery ratio using a weight *w* to prioritise one of these values over the other. Each value calculated here = (1 − *w*)*PayoffRatio* + (*w*)*RecoveryRate*. Left to right, *w* = [0.0, 0.25, 0.5, 0.75, 1.0]. Plots produced via Jupyter notebook.

With all of the results gathered from the computation model, we also consider the potential trade off between prosperity and stability within these networks. This is illustrated in Fig 11 where both of these factors are weighted against each other as different network rankings are utilised. When considering only the potential payoffs of individuals, ranking methods such as degree highest and eigenvector highest fare the best with significantly more average payoff than other networks, regardless of information prioritisation (see Fig 11). Rankings where less connected individuals are favoured generally do not attain as much payoff between individuals, particularly degree low and eigenvector low, which is due to significantly less connections being formed amongst individuals. However, as stability becomes a more favoured factor, the effectiveness of degree high and eigenvector high is significantly diminished as these prove highly ineffective at avoiding successful cheater invasions. Here, other methods such as degree low and eigenvector low emerge as offering significantly better resilience to cheater invasion, which can also be attributed to low connectivity (see Fig 11). When considering both of these factors, betweeness emerges as one of the generally better methods of ranking, as it offers strong network resilience to cheater whilst also allowing for some payoff to be cultivated amongst individuals.

## 4 Discussion

By utilising a novel computation model where newcomers make use of network rankings as part of their decision-making process, we have been able to illustrate this choice of ranking as public information can have an effect on cooperative complex networks. Particularly, the resilience of these networks against cheater invasion and how changes to network topology can also effect the payoffs cultivated by cooperative individuals before an invasion.

One of the key observations from the results is that the choice of ranking utilised by newcomers to serve as public information can have a significant effect on the likelihood of fending off cheater invasions, especially when the chosen method does not favour more interconnected individuals. In previous works [15, 17], public information is modelled as indicating positively when an individual’s connection count exceeds that of the network average. This is considered as the degree highest (Deg. hi) method within the context of the model utilised here. Like these previous works [15, 17], once *τ* exceeds a certain point, collapses in cooperative networks become far more commonplace (see Figs 6a, 8a & 10a), which can be attributed to the abundance of connections serving as the ideal conditions for a cheater invasion to be successful (see Figs 4, 6, 8 & 10). While individuals benefit greatly for a period under these circumstances due to a significant gain to payoffs (see Figs 4b, 6b, 8b & 10b), these gains are rendered moot in the long run where successful invasions will ultimately result in all individuals receiving no payoff, a very possible occurrence under these conditions (see Fig 11).

**Fig 12:**
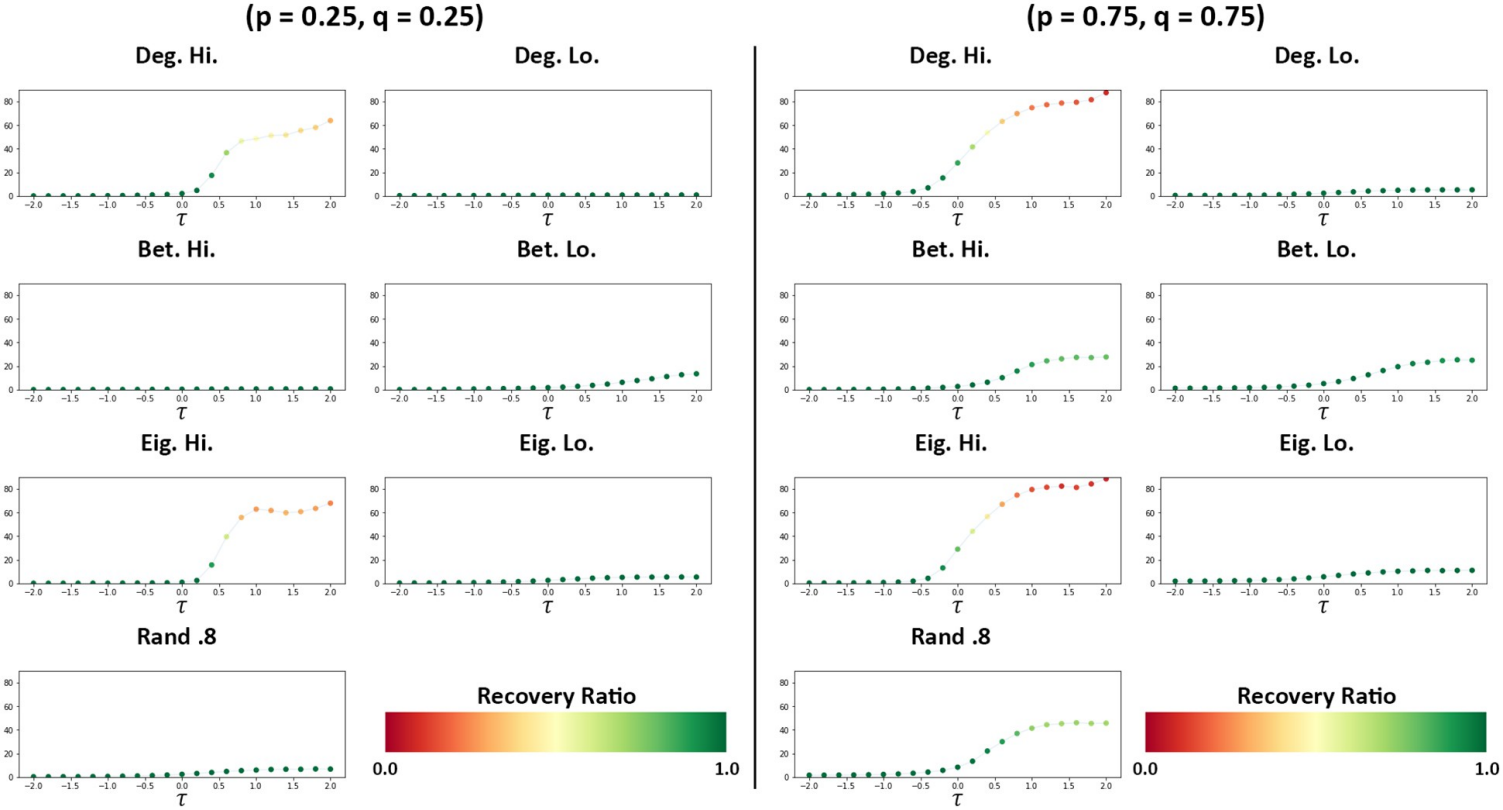
The choice of network metric employed as public information can lead to noticeable gains in payoffs amongst individuals at an increased risk of successful cheater invasion. Depending on the strength of information signals, methods such as betweeness can enable individuals to cultivate some degree of payoff amongst each other whilst avoiding any significant increases in the number of observed cooperative collapses. Each plot visualises the average payoff of individuals at the point just before an induced cheater invasion against the recovery ratio, which is illustrated here on a color scale. *τ* represents the decision threshold. Plots produced via Jupyter Notebook.

As illustrated in the results, this is something that cannot realistically be addressed by simply considering the inverse of only favouring individuals with low connection counts. In the case of degree lowest (Deg. lo), while these networks are highly resistant to cheater invasion (see Figs 4a, 6a, 8a & 10a), this does come at the significant cost of very little payoff being cultivated by cooperative individuals (see Figs 4b, 6b, 8b & 10b). Networks may be capable of avoiding networks being taken over by cheaters, but as a consequence, individuals will be contributing very little to the network due to little connectivity (see Figs 4, 6, 8 & 10). In Fig 11, the trade-off between stability and prosperity is explored. Whilst some rankings offer ways of cultivating significantly higher individual payoffs, this can be greatly impacted when also considering the general stability of networks (see Figs 11 and 12). Whilst high payoffs can be attained by individuals for a time, but this will likely lead to significant and costly disruption in the long term or networks can be highly resilient against cheater invasion at a significant cost to overall individual contribution to the well-being of these networks.

Of the methods evaluated as part of this model, betweeness strikes some balance here (see Figs 11 and 12). In the networks where betweeness is utilised by newcomers, these typically result in some moderate gains to overall payoff (see Figs 4b, 6b, 8b & 10b) with a minor cost to potential recovery rates (see Figs 4a, 6a, 8a & 10a). Some reasoning for this could be how the usage of both of these rankings leads to some increases to connectivity whilst leaning towards a lower level of connectivity per individual within the network (see Figs 4, 6, 8 & 10). These network configurations seemed to have led to more isolated communities of individuals, where they can make moderate gains in terms of payoff, whilst also avoiding the very high level of connectivity that are the ideal conditions for cheater invasions. While the random method (Rand .8) avoids the most significant drops in recoveries, networks using this are mired by noticeably reduced recovery times (see Figs 4c, 6c, 8c & 10c). The random method also results in an increase in connectivity (see Fig 5) but does not seem to have strong influence in kerbing the number of connections present in these networks. These suggest that the random method may be a somewhat less reliable choice that other rankings in terms of managing network connectivity.

These proposed methods to collect public information could be utilised differently throughout the life cycle of these networks in order to more properly utilise them. The proposed rankings could be utilised with the aid of bots for example, following the the work carried out by Shirado et al. [12]. The researchers examined the use of bots to aid with individual decision making during a public goods game. These bots would aid with allowing participants to rewire their connections with others, which could be based on their neighbours chosen strategies. Of the bot strategies employed, the most reliable appeared to be one where bots would offer different rewiring options based on what occurred in the previous round. For example, if someone’s neighbour cheated in the previous round, the bot would opt to suggest removing that connection rather than suggest creating a new connection with another cooperative individual if that didn’t occur. It may warrant further investigation to consider how adjusting how particular rankings are utilised at various points in a network could also benefit the well-being and reliance of these networks. Other network rankings and new proposed methods should also be considered in future works in order to determine how newcomers in these networks can be better informed when determining who to associate with. These methods should also take any required processing time and resources into account, some of the rankings utilised here, particularly betweeness and eigenvector, had a significant impact to processing these simulations and it is likely that the computational cost would greatly increase with networks of populations that exceed *N* = 100. Different network structures of varying sizes should also be considered as part of future works in order to determine the effects of these methods in those scenarios. Here, Erdős-Réyni was utilised to initialise the starting structure of the network, where in future works, methods such as ring and scale-free networks should also be considered. The use of these network rankings and their potential effects should also be considered against real-world network structures to further determine how they could potentially aid individuals, a starting point for this could be based upon social networks such as x.com.

Lastly, although the focus of this paper is to consider different methods to serve as public information to newcomers, the findings here also emphasize the importance that individual private information can have within these networks. As already highlighted in Figs 6 and 10, when newcomers utilise a stronger signal from private information (*q* = 0.75), a significant increase to connectivity occurs, particularly when compared with networks where only public information is prioritised (*p* = 0.75). When newcomers rely mostly on public information, they become less likely to form connections, which feeds back into available public information and suggests that this discourages too many connections being formed between individuals. A similar occurrence was found in previous work [11], where networks stagnated in terms of cultivated cooperation, avoiding any sharp collapses but otherwise resulting in little payoff between individuals. While in future works the proposed questions and scenarios may revolve around what additional information and resources could be provided to individuals within these networks, private information should also continue to be considered when examining the dynamics of these networks as it appears to be a primary driver of encouraging individual agency.

Overall, the findings of this paper illustrates how the ability to rank potential partners using standard network rankings can help to control the trade-off between prosperity and stability (see Figs 11 and 12). Of the proposed rankings, betweeness emerges as one of the more reliable methods for aiding with the resilience of networks whilst allowing for some room for individuals to cooperate with their associated neighbours. As part of future work, more refined approaches to utilising these rankings should be considered as well as considering other rankings and methods for evaluating potential neighbours, particularly those that are computationally efficient [23].
